# A distinct IgM immunoblot signature differentiates neurosyphilis from latent syphilis

**DOI:** 10.1128/spectrum.04141-25

**Published:** 2026-04-30

**Authors:** Xue Yang, Jiahui Shen, Ming Liang, Ping Luo, Donghua Liu

**Affiliations:** 1 Department of Dermatology and Venereology, First Affiliated Hospital of Guangxi Medical University 687412, Nanning, People's Republic of China; Tainan Hospital Ministry of Health and Welfare, Tainan, Taiwan

**Keywords:** neurosyphilis, latent syphilis, immunoblot, IgM, diagnosis

## Abstract

Accurately differentiating neurosyphilis (NS) from latent syphilis (LS) is essential, as it directly guides therapeutic decisions and prevents devastating neurological complications. However, this differentiation remains a significant clinical challenge because it currently relies on cerebrospinal fluid analysis—an invasive and resource-intensive procedure that is not suitable for all patients. To address this, we evaluated whether a detailed *Treponema pallidum*-specific immunoglobulin M (IgM) immunoblot profile could offer a robust non-invasive diagnostic tool. We conducted a comprehensive analysis of serum samples from 319 patients (74 NS and 245 LS), assessing the IgM positive rate, antigen-specific reactivity (TP15, TP17, TP45, and TP47), and antibody reaction intensity (gray value). Our analysis revealed that NS patients exhibited a significantly stronger and broader IgM response, evidenced by a higher overall positive rate (45.9% vs 26.1%, *P* = 0.001) and elevated reactivity to TP17, TP45, and TP47 antigens. The antibody reaction intensity for these antigens was also significantly stronger in the NS group. Critically, multivariable analysis identified a higher serum the Toluidine Red Unheated Serum Test titer (odds Ratio [OR] = 1.025 per unit, 95% confidence interval [CI]: 1.009–1.041, *P* = 0.003) and a greater number of positive antigens (OR = 1.663, 95% CI: 1.100–2.512, *P* = 0.016) as independent risk factors for NS, while male sex was an independent protective factor (OR = 0.325, 95% CI: 0.179–0.591, *P* < 0.001). These findings demonstrate that the IgM immunoblot provides a distinct serological signature, offering a valuable non-invasive aid for discriminating NS from LS.

**IMPORTANCE:**

This study addresses a fundamental challenge in syphilis management: determining which patients with latent syphilis are at high risk for neurosyphilis without relying on invasive lumbar puncture. By analyzing a simple blood test that profiles the immunoglobulin M antibody response in detail, we discovered that patients with neurosyphilis have a distinctly stronger and broader immune reaction. We further identified key risk factors, including higher antibody levels and reactivity to multiple targets. These findings are significant because they provide doctors with a practical, non-invasive tool to stratify patient risk more accurately. This can help optimize clinical decision-making, ensuring that lumbar punctures are performed on patients who need them most while avoiding unnecessary procedures for others, ultimately leading to better and more personalized patient care.

## INTRODUCTION

Neurosyphilis (NS), the infection of the central nervous system by *Treponema pallidum*, represents a severe manifestation of syphilis with potentially devastating neurological consequences. Its clinical presentation is notoriously heterogeneous, ranging from asymptomatic to severe neuropsychiatric decline (1). Latent syphilis (LS) refers to an asymptomatic stage of infection characterized by positive serological tests in the absence of clear clinical manifestations. It is categorized as early latent (<1 year after infection) or late latent (>1 year after infection or of unknown duration) (2). There is no consensus on when to perform a lumbar puncture to rule out neurosyphilis in patients with latent syphilis, and clinical decision-making often relies heavily on individual risk assessment. (3, 4) Consequently, there is an urgent need for robust serological biomarkers that can accurately identify patients at high risk for NS, thereby optimizing the use of lumbar puncture.

The immunoglobulin M (IgM) antibody response, as the first line of specific humoral immunity, is a promising indicator of active or recent infection. In syphilis, the presence of *Treponema pallidum*-specific IgM has been postulated to correlate with disease activity (5, 6). However, conventional serological tests provide a limited, often qualitative, assessment. By simultaneously evaluating the antibody response against multiple recombinant antigens (e.g., TP15, TP17, TP45, and TP47), the IgM immunoblot technique affords a more comprehensive assessment of the host immune response, capturing both qualitative and quantitative information not available from single-parameter assays. We hypothesize that neuroinvasive infection elicits a quantitatively and qualitatively distinct systemic IgM response compared to latent infection.

This study aimed first to determine whether a quantitative IgM immunoblot profile provides discriminatory value between latent syphilis and neurosyphilis. The second was to integrate this profile into a multivariable model to identify independent risk factors for neurosyphilis and objectively stratify patient risk.

## MATERIALS AND METHODS

### Diagnostic criteria

Latent syphilis was defined as an asymptomatic infection, confirmed by a positive Toluidine Red Unheated Serum Test (TRUST) and a positive *Treponema pallidum* particle agglutination (TPPA) test, in the absence of cutaneous, mucosal, or neurological manifestations. It was further categorized as early latent syphilis if the infection was acquired within the preceding 1 year, and as late latent syphilis if the duration exceeded 1 year or could not be ascertained.

NS was diagnosed based on the following criteria (7–11): Symptomatic NS: Diagnosed if neurological symptoms/signs (e.g., headache, psychiatric abnormalities, and cognitive decline) were present and at least one supportive cerebrospinal fluid (CSF) abnormality was confirmed: (i) a positive CSF-TPPA; or (ii) both elevated CSF protein (>450 mg/L) and CSF-white blood cell (WBC) (>5/µL). Asymptomatic NS: Diagnosed if neurological symptoms/signs were absent and all of the following CSF criteria were met: a positive CSF-TPPA, elevated CSF protein (>450 mg/L), and CSF-WBC > 5/µL.

### Study population

This retrospective cohort study was conducted at the First Affiliated Hospital of Guangxi Medical University. The study population comprised patients diagnosed with either latent syphilis or neurosyphilis between January 2024 and October 2025, who were identified through a search of the hospital’s electronic medical record system. Key exclusion criteria were applied: (i) documented co-infection with HIV; (ii) incomplete or ambiguous clinical, serological, or cerebrospinal fluid data required for definitive classification; (iii) a documented history of adequate antisyphilitic treatment within the preceding year. All relevant data, including demographic characteristics, clinical presentation, serological test results (TRUST, TPPA, and TP-IgM immunoblot), and cerebrospinal fluid analysis findings, were systematically extracted from the hospital’s integrated electronic medical record and laboratory information management systems.

### TP-IgM immunoblot assay

The *Treponema pallidum*-specific IgM antibody profile was detected using the commercial EUROLINE-WB (IgM) kit (EUROIMMUN, Germany) according to the manufacturer’s instructions. This immunoassay uses a membrane strip coated with electrophoretically separated *Treponema pallidum* antigens, including TP15, TP17, TP45, and TP47. Briefly, patient sera were diluted 1:51 with the provided universal buffer. The diluted serum (1.5 mL) was incubated with the antigen-coated membrane strip for 30 min at room temperature on a rocking platform. After washing, the strips were incubated with an alkaline phosphatase-conjugated goat anti-human IgM antibody (diluted 1:10) for an additional 30 min. Following a second washing step, the antigen-antibody complexes were visualized by adding the substrate solution (NBT/BCIP) for 10 min. The reaction was stopped by rinsing with distilled water.

### Interpretation of immunoblot results

The developed strips were air-dried and scanned for quantitative analysis. The intensity of each antigen-specific band was measured as a gray value using image analysis software (EUROLineScan). For quantitative analysis, which was the focus of this study, a gray value threshold of >14 was set as a positive reaction for each antigen. The number of positive antigens (ranging from 0 to 4) was also calculated for each patient.

### Statistical analysis

All statistical analyses were performed using SPSS software, version 24.0. Continuous variables were presented as median and interquartile range (IQR) due to non-normal distribution, and comparisons between groups were made using the Mann-Whitney *U* test. Categorical variables were presented as numbers (percentages) and compared using the *χ*^2^ test or Fisher’s exact test as appropriate. A *P* value < 0.05 (two-tailed) was considered statistically significant.

## RESULTS

A total of 319 patients with syphilis were included in this study, comprising 74 patients with NS and 245 patients with LS, which included 84 with early latent and 161 with late latent syphilis. The baseline demographic and clinical characteristics of the two groups are summarized in Table 1.

**TABLE 1:** General clinical information of 319 patients with syphilis^*a*^

Variable	Latent^*b*^ (*n* = 245)	Neurosyphilis (*n* = 74)	*P* value
Sex, male, *n* (%)	110 (44.9%)	52 (70.3%)	0.001
Age (median, IQR), years	40.0 (28.5–56.5）	41.0 (29.0–56.0)	0.790
Blood-reactive TPPA, *n* (%)	245 (100%)	74 (100%)	–^*d*^
Blood-reactive TRUST, *n* (%)	245 (100%)	74 (100%)	–
1/Serum TRUST titer (median, IQR)	2 (1–8）	16 (4–32)	<0.001
CSF protein, mg/L (median, IQR)	307.4 (237.7–392.2)^*c*^	419.9 (322.6–540.7)	0.002
CSF WBC, μL (median, IQR)	2 (0–5.3)^*c*^	5 (2–20)	0.004
CSF TPPA (+), *n* (%)	0 (0.0%)^*c*^	69 (93.2%)	<0.001
CSF TRUST (+), *n* (%)	0 (0.0%)^*c*^	27 (36.5%)	<0.001

^*a*^ CSF, cerebrospinal fluid; IQR, interquartile range; TPPA, *Treponema pallidum* particle agglutination; TRUST, Toluidine Red Unheated Serum Test; WBC, white blood cell.

^*b*^ Included 84 early latent syphilis and 161 late latent syphilis.

^*c*^ CSF parameters were available and analyzed for 30 LS patients (*n* = 30) who had undergone lumbar puncture.

^*d*^ "–" indicates that a statistical comparison was not applicable, as both groups exhibited identical 100% positive rates.

### Neurosyphilis is associated with a stronger and broader IgM immunoblot response

**Fig 1:**
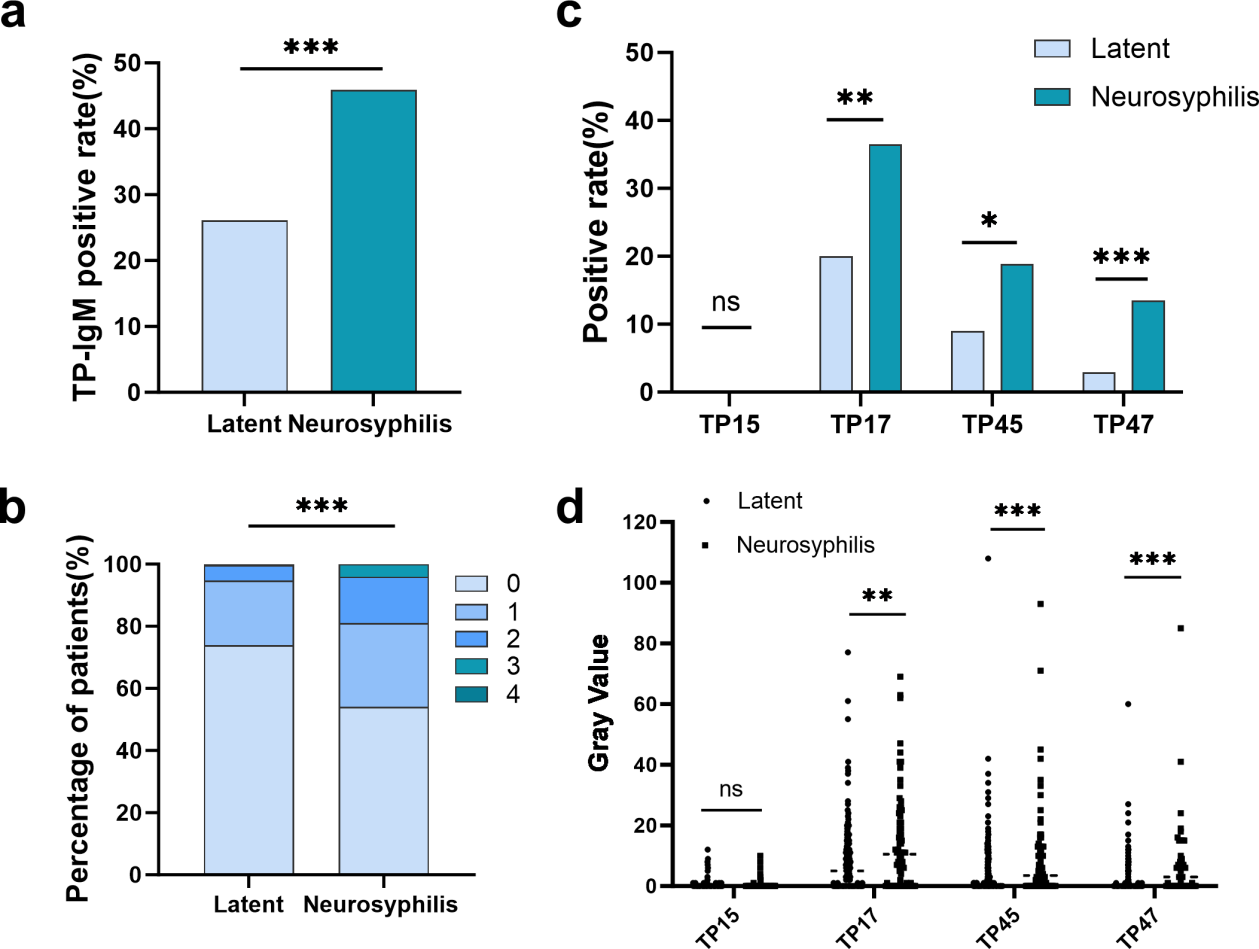
Comparison of *Treponema pallidum*-specific IgM immunoblot profiles between patients with latent syphilis (*n* = 245) and neurosyphilis (*n* = 74). (**a**) The overall IgM positivity rate was significantly higher in the neurosyphilis group compared to the latent syphilis group. (**b**) The distribution pattern of the number of reactive antigen bands (0–4) differed between the two groups, with a higher proportion of NS patients recognizing multiple antigens. (**c**) Antigen-specific positive rates for TP17, TP45, and TP47 were significantly elevated in the NS group. No reactivity to TP15 was observed. (**d**) The antibody reaction intensity (gray value) for TP17, TP45, and TP47 was significantly stronger in the neurosyphilis group among IgM-positive patients. Data are presented as percentages (**a–c**) or scatter plots with medians (**d**). *P < 0.05, **P < 0.01, ***P < 0.001; ns, not significant.

A marked disparity in the serum TP-IgM immunoblot profile was observed between patients with LS and NS. The overall IgM positive rate was significantly elevated in the NS group compared to the LS group (45.9% vs 26.1%; *P* = 0.001, Fig. 1a). The distribution of the number of reactive antigens per patient further underscored this difference, with a significantly greater proportion of NS patients recognizing multiple (≥2) antigens (Fig. 1b). This enhanced serological response in NS patients was driven by significantly higher positive rates for the TP17 (36.5% vs 20.0%), TP45 (18.9% vs 9.0%), and TP47 (13.5% vs 2.9%) antigens (all *P* < 0.05, Fig. 1c). In contrast, the TP15 antigen was uniformly negative across all patients in both cohorts. Furthermore, the antibody reaction intensity was significantly stronger in the NS group. Specifically, the median gray value for TP17 was 10.5 (IQR 0–21.5) in NS patients vs 5.0 (IQR 0–12) in LS patients (*P* = 0.002). For TP45, the median was 3.5 (IQR 0–13) vs 0 (IQR 0–7.0; *P* < 0.001), and for TP47, it was 3.0 (IQR 0–12.5) vs 0 (IQR 0–0; *P* < 0.001). In contrast, for TP15, the median gray value was 0 (IQR 0–0) in both groups, with no significant difference observed (*P* > 0.05; Fig. 1d).

### Multivariable logistic regression analysis for neurosyphilis predictors

A multivariable logistic regression analysis was performed to identify factors independently associated with NS. The variable selection process was conducted to refine the model and assess the contribution of different aspects of the IgM response. Using the forward likelihood ratio method from a candidate set that included (i) demographic factors (age and sex), (ii) conventional serological activity (serum TRUST titer), and (iii) TP-IgM immunoblot profiles—encompassing both global measures (the overall TP-IgM reactivity [positive/negative] and the number of positive IgM bands) and detailed measures (antigen-specific reactivity [positive/negative for TP17, TP45, TP47] and their respective reaction intensity [gray value])—the final model identified the most parsimonious set of predictors. The final model identified three significant independent predictors (Table 2). Male sex was an important protective factor (OR = 0.325, 95% CI: 0.179–0.591, *P* < 0.001). In contrast, a higher serum TRUST titer (OR = 1.025 per unit increase, 95% CI: 1.009–1.041, *P* = 0.003) and a greater number of positive IgM bands (OR = 1.663, 95% CI: 1.100–2.512, *P* = 0.016) were independent risk factors. The model demonstrated a good fit (Nagelkerke *R*² = 0.201) and classification accuracy of 76.8%, with no evidence of multicollinearity (all variance inflation factors < 2.0).

**TABLE 2:** Multivariable logistic regression analysis of predictors for neurosyphilis (*n* = 319)^*a*^^,*b*^

Variate	β	SE (β)	Wald	*P*	OR (95% CI)
Demographic factor					
Sex (male)^*c*^	−1.124	0.305	13.604	<0.001	0.325 (0.179–0.591)
Serological markers					
Serum TRUST titer (per unit)	0.024	0.008	9.119	0.003	1.025 (1.009–1.041)
Number of positive IgM bands	0.509	0.211	5.830	0.016	1.663 (1.100–2.512)
Constant (*C*)	−1.254	0.206	36.934	<0.001	0.285

^*a*^ The analysis was based on the total cohort of 319 patients (74 neurosyphilis and 245 latent syphilis).

^*b*^ OR, odds ratio; CI, confidence interval.

^*c*^ The reference category for Sex is Female.

## DISCUSSION

Neurosyphilis, caused by *Treponema pallidum* invasion of the central nervous system, may manifest as diverse psychiatric syndromes, including depression, mania, psychosis, personality changes, delirium, and dementia, particularly in untreated cases (12–14). A study demonstrated a neurosyphilis prevalence rate of 26.6% among patients with latent syphilis (3). Given this significant disease burden, accurately differentiating between NS and LS is critically important, as it not only helps avert irreversible neurological damage but also determines the appropriate treatment regimen. Specifically, patients diagnosed with neurosyphilis typically require more intensive therapy, such as intravenous aqueous crystalline penicillin G at 18–24 million units daily (administered as 3–4 million units every 4 h) for 10–14 days, often followed by a course of intramuscular benzathine penicillin G (2.4 million units once weekly for three doses) to eradicate any residual systemic infection. In contrast, patients with latent syphilis are adequately treated with intramuscular benzathine penicillin G at a dose of 2.4 million units, administered once weekly for three doses (15, 16). The identification of latent syphilis patients at risk of progressing to neurosyphilis in the absence of lumbar puncture remains a persistent clinical challenge. (4) Current research indicates that serological biomarkers capable of distinguishing latent from neurosyphilis include conventional markers such as the serum rapid plasma reagin titer (17), along with novel candidates like the chemokine CXCL13 (18), proteins associated with neuronal damage, including GFAP, NfL, and UCH-L1 (19), and cytokines such as IL-26 (20). Given this array of novel biomarkers, our study specifically focused on evaluating the diagnostic potential of the IgM immunoblot, a more nuanced serological tool, for neurosyphilis.

Our study demonstrates that patients with neurosyphilis exhibit a quantitatively and qualitatively distinct humoral immune response compared to those with latent syphilis, characterized by a higher frequency and intensity of IgM reactivity against specific *T. pallidum* antigens, particularly TP47. The observed enhancement in both the breadth and intensity of the IgM response in neurosyphilis patients is biologically plausible. This enhanced response can be attributed to the persistence of *T. pallidum* within the central nervous system, which provides a sustained antigenic stimulus that drives a more robust systemic antibody production. Furthermore, this intrathecal synthesis is directly evidenced by prior findings that an elevated CSF/serum IgM index, corrected for albumin, serves as a key indicator of central nervous system infection (21). The prominent role of the TP47 antigen, a major membrane immunogen, underscores its high immunogenicity and potential as a key biomarker for neuroinvasive disease. This finding complements previous studies suggesting diagnostic utility for anti-TP47 antibodies in neurosyphilis. A study revealed TP47 as a key factor in neurosyphilis by inducing microglial cell death and driving the disease’s pathogenesis (22). Accordingly, previous studies have further established the diagnostic potential of serum antibodies against TP47 and TP17, demonstrating their promising capability and ability to enhance predictive accuracy for NS diagnosis (23). Notwithstanding this established potential, our findings reveal that a high proportion of neurosyphilis patients (54.1%) test negative by IgM immunoblot, indicating that a negative result cannot rule out the disease. Potential reasons include a waned IgM response in late-stage infection (24), a compartmentalized central nervous system immune response not fully reflected in serum, or individual variations in immunity and pathogen strain (1, 25). Therefore, the test’s clinical value lies not in exclusion but in the predictive power of a positive result. A positive immunoblot and a high serum TRUST titer indicate a high-risk serological profile that warrants further investigation.

Our study aimed to identify which specific components of the TP-IgM immunoblot profile hold independent value for distinguishing NS from LS. To achieve this, we performed a multivariable logistic regression, which established that a higher serum TRUST titer and a greater number of reactive antigens were independent risk factors, while male sex was a protective factor. The finding of male sex as an independent protective factor contrasts with several earlier studies that reported male sex as a risk factor (17, 26). While the underlying reasons remain unclear and should be interpreted with caution, a plausible explanation might be that females require a higher threshold of serological activity to develop neurological involvement, potentially suggesting a sexually dimorphic immune response. Alternatively, differences in healthcare-seeking behavior or diagnostic vigilance could also be contributing factors. Therefore, this association warrants further investigation to elucidate its precise biological or behavioral mechanisms. The prognostic significance of the quantitative serum TRUST titer for NS is well established (27, 28), with a robust association observed between titers ≥1:16 and an increased odds of neurosyphilis (29). Furthermore, the number of reactive antigens on the IgM immunoblot, which indicates the breadth of the immune response, was an independent predictor of neurosyphilis. A graded relationship was observed, whereby each additional positive band significantly increased the odds of neurosyphilis, suggesting a cumulative effect of the serological response. Consequently, based on our data, the combination of a high TRUST titer and multiple reactive antigens (≥2 bands) helps to identify a high-risk serological profile. We therefore suggest that lumbar puncture should be strongly considered for patients with latent syphilis who meet these criteria, as an adjunct to clinical judgment.

The main limitations of this study are its cross-sectional design and the use of specific recombinant antigens. First, the retrospective design establishes an association but cannot demonstrate causality or differentiate whether the observed IgM signature reflects neuroinvasion vs recent systemic relapse. Therefore, prospective studies are needed to validate its predictive value for progression to neurosyphilis. Second, while the recombinant antigens (TP15, TP17, TP45, and TP47) are relatively conserved, the potential influence of *Treponema pallidum* strain variation on the signature’s performance warrants investigation in future multi-center, geographically diverse cohorts.

In conclusion, this study indicates that a positive IgM immunoblot, particularly with multiple reactive bands and a high serum TRUST titer, constitutes a high-risk serological signature for neurosyphilis. The presence of this signature in patients with latent syphilis should prompt strong consideration for lumbar puncture. Future prospective and multi-center studies are warranted to validate the clinical utility of this signature and refine its application.
